# Engineered protein nanoclusters reduce liver fibrosis and hepatocellular carcinoma in mice models

**DOI:** 10.1016/j.bioactmat.2026.05.038

**Published:** 2026-05-27

**Authors:** Tanya Saxena, Gabriela Guedes, Inês Domingues, Hafsa Yagoubi, Léo Guilbaud, William Van den Bossche, Cristina Pangua, Greetje Vande Velde, Bernard Ucakar, Andrea García-Esnaola, Isabelle Leclercq, Aitziber L. Cortajarena, Ana Beloqui

**Affiliations:** a UCLouvain, Louvain Drug Research Institute, Advanced Drug Delivery and Biomaterials, Brussels, 1200, Belgium; b Center for Cooperative Research in Biomaterials (CIC biomaGUNE), Basque Research and Technology Alliance (BRTA), Paseo de Miramón 194, Donostia-San Sebastián, 20014, Spain; c Biomedical MRI Unit, Department of Imaging and Pathology, KU Leuven, Leuven, 3000, Belgium; d UCLouvain, Institute of Experimental and Clinical Research, Laboratory of Hepato-Gastroenterology, Avenue Emmanuel Mounier 53, Brussels, 1200, Belgium; e Ikerbasque. Basque Foundation for Science, Bilbao, 48009, Spain; f WEL Research Institute, avenue Pasteur, 6, Wavre, 1300, Belgium

**Keywords:** Anti-fibrotic therapy, Engineered protein therapeutics, Hepatocellular carcinoma, Liver fibrosis, Protein–nanomaterial hybrids

## Abstract

Long-term liver injury leads to acute hepatitis and chronic liver diseases, including fibrosis and hepatocellular carcinoma (HCC), which frequently necessitate liver transplantation and are associated with poor median survival. Transforming growth factor-β (TGF-β) signalling pathway is a key driver of fibrogenesis and tumor progression. Therapeutic approaches targeting this pathway—via TGF-β receptor blockade or inhibition of its chaperone protein Hsp90— are under pre-clinical and clinical trials, and despite some promising results none of them has been adopted as a definitive therapy. Engineered protein scaffolds have emerged as an attractive alternative owing to their high engineerability and ease of production. In particular, consensus tetratricopeptide repeat (CTPR) proteins are strong candidates because of their modularity, robustness, design flexibility, and amenability to functionalization. Here, we investigate the protein–nanocluster hybrid formulation comprising C390 and nanocluster-stabilizing domain (C390-AuNC) as a Hsp90 inhibitory platform to assess its potential dual anti-fibrotic and anti-tumor activity in two chemically-induced murine liver disease models: acute liver fibrosis and chronic HCC. In the fibrosis model, C390-AuNC markedly suppress the expression of profibrotic markers and promote degradation of collagen fibers in the liver, indicating effective attenuation of fibrogenesis. In the HCC model, C390-AuNC inhibit Hsp90, leading to reduced expression of oncogenic proteins that drive cancer cell proliferation and metastasis, and consequently diminish tumor burden. Collectively, these findings support C390-AuNC as a promising next-generation biotherapeutic platform with low anticipated immunogenicity and high therapeutic potential.

## Highlights

• Engineered proteins like C390 mimic naturally occurring domains showing high binding affinity for Hsp90 protein.
• I.p. administered C390-gold nanoclusters (C390-AuNC) exhibit strong liver targeting and negligible side effects.
• C390-AuNC downregulate TGF-β signaling, reducing collagen synthesis and liver fibrosis.
• Twice-weekly C390-AuNC reduced tumor burden, lowering cell proliferation (Ki67) and pro-metastatic (MMP-2) markers.

## Graphical abstract

**Figure:**
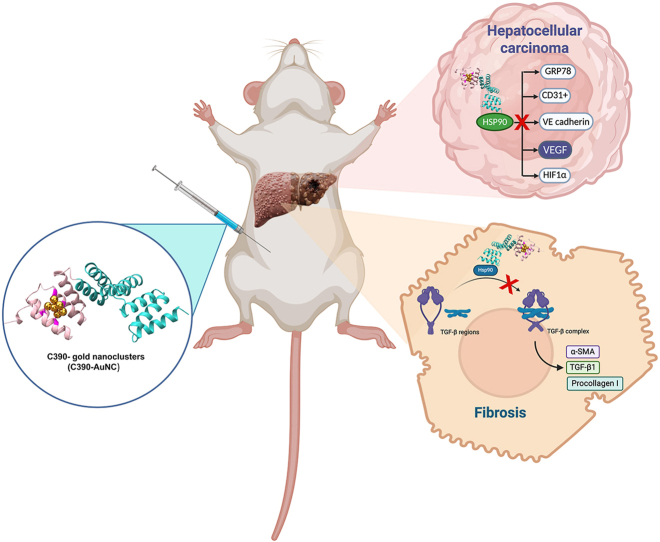

## 1 Introduction

Liver injury is caused due to chemicals, dietary lifestyle or genetic factors [1]. Approximately 3.3% of the world's population suffers from advanced liver fibrosis consequently leading to 1.3% suffering from liver cirrhosis [2]. As per World Health Organization, 830,000 global deaths were reported due to hepatobiliary cancer or hepatocellular carcinoma (HCC) in 2020 [3]. The severity of diseased conditions in the liver, progress from an acute and reversible inflammation/hepatitis to more chronic conditions like fibrosis, cirrhosis and liver cancer characterized by accumulation of collagen, scarred tissue initially, to eventually tumor development and liver failure in advanced stages [4]. Clinical treatments include lifestyle changes or etiological treatment to prevent or reverse initial liver fibrosis, symptomatic treatment in cirrhosis, and tyrosine kinase inhibitors (TKIs) like lenvatinib or radiation therapy to treat HCC, providing merely an increased median survival (up to 10 months with TKIs) [5]. Liver transplant is the ultimate curative treatment for advanced and decompensated liver diseases [6].

Currently, there is no direct anti-fibrotic treatment approved for liver disease. Over the past years, considerable effort has been devoted to developing therapeutic strategies targeting the transforming growth factor-β (TGF-β) pathway due to its central role in fibrosis and cancer progression as shown in Schematic 1 [9,10]. Despite these efforts, the only FDA-approved TGF-β modulator is liuspatercept, prescribed for anemia in adults with myelodysplastic syndromes (MDS). Several other TGF-β targeted therapies are in pre-clinical development, especially for HCC. For example, galunisertib, a small molecule inhibitor of the TGF-βR1, has shown minimal side effects and improved overall survival in patients with low levels of alpha-fetoprotein (AFP) [11]. Likewise, NIS793, a neutralizing antibody, binds to active TGF-β in patients, reducing its circulating levels [12], while livmoniplimab, a humanized monoclonal antibody targeting the LRRC32/TGF-β1 complex, exhibits positive efficacy in combination with PD-1 inhibition in a phase III clinical trial for advanced HCC (NCT06109272) [13].

**Schematic 1:**
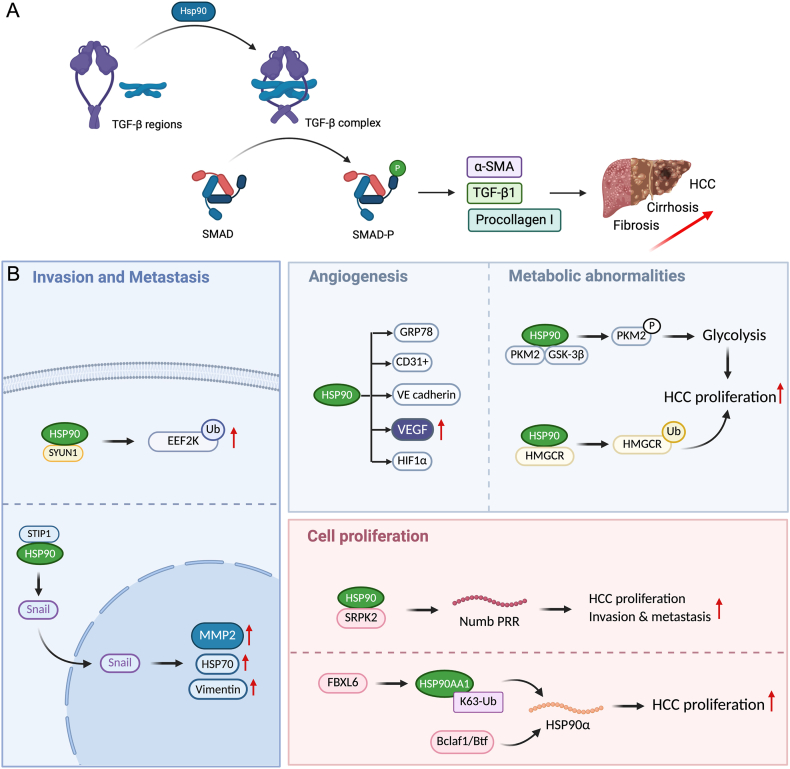
Hsp90-mediated liver fibrosis and HCC pathways. (A) Activation of TGF-β complex by Hsp90 protein leading to release of α-SMA, TGF-β1 and Procollagen 1 (Col1α) which generate liver fibrosis, cirrhosis and eventually HCC. (B) Other cellular pathways stimulated by Hsp90 that lead to angiogenesis, cell proliferation and metastasis that cause tumor growth in HCC [7,8].

Despite the promising results observed with these TGF-β targeted therapies, none of them have been widely adopted as a liver fibrosis or HCC therapy in clinical practice. This is likely due to the fundamental and ubiquitous role of TGF-β in wound healing and tissue homeostasis. Consequently, the systemic and prolonged inhibition of TGF-β may result in severe adverse effects like bleeding and cardiotoxicity. Besides, these treatments mainly rely on combination therapy with antibodies, which involve costly and complex production, poor batch-to-batch reproducibility, and pose the risk of immune reactions [14,15]. One promising alternative to TGF-β targeting is the inhibition of the molecular chaperone heat shock protein 90 (Hsp 90) that converts inactive TGF-β regions into active TGF-β complex in the cell as illustrated in Schematic 1A [16]. The inhibition of Hsp90 destabilizes TGF-β signalling cascade, resulting in the downregulation of the TGF-β pathway and downstream profibrotic effects, including the reduction in collagen synthesis and a consequent decrease in fibrosis [17].

Beyond its role in TGF-β signalling, Hsp90 helps in maintaining cell homeostasis by remodelling extracellular matrix and preventing misfolding of crucial proteins [18]. However, in diseased states such as cancer, it prevents apoptosis of cancer cells and is upregulated by 3-fold leading to faster cell proliferation, tumor growth, and metastasis [7]. Thus, Hsp90 inhibitors can potentially act as anti-fibrotic as well as anti-cancerous agents. Currently, more than 20 Hsp90 inhibitors have entered clinical trials for anti-tumor effects, but there is only one commercially available Hsp90 inhibitor named Pimitespib (TAS-116), which is approved in Japan for treating gastrointestinal stromal tumors [19]. However, its previous analogs have shown long-term severe side effects and better alternatives are being investigated in pre-clinical research [20].

In addition to engineered antibodies, designed protein therapeutics are emerging as attractive alternatives to small molecule inhibitors [21]. These engineered proteins typically exploit and target naturally occurring protein-protein interactions. They can be precisely engineered to achieve high affinity and specificity for targets, enabling tailored modulatory activities with reduced off-target effects. In particular, engineered proteins inspired by naturally occurring repeat proteins have been widely exploited as a scaffold for the development of new protein biologics, binding with high affinity and selectivity to their targets [22]. Among these, consensus tetratricopeptide repeat (CTPR) proteins stand out for their robustness, modularity, and design flexibility. The CTPR unit constitutes of 34 amino acids, of which only eight are highly conserved and essential to ensure proper folding [23], providing extensive mutational flexibility. This high tolerance to sequence variation enabled the design of a CTPR-based domain that binds the C-terminal region of Hsp90, named the C390 module [24]. This module has previously shown therapeutic efficacy against breast cancer [25] and cardiac fibrosis [17]. In the context of cardiac fibrosis, C390 was found to promote the disruption of the TGF-β-Hsp90 complex, resulting in a significant decrease in fibrosis and heart hypertrophy *in vivo* [26]. Importantly, inhibition by C390 maintained the chaperone activity of Hsp90, minimizing the deleterious effects on cellular homeostasis thereby avoiding the adverse effects commonly observed for other Hsp90 inhibitors.

Building on the modularity and mutation permissibility of CTPR proteins, these modules can also be engineered to display metal-coordination sites that allow stabilization of metal nanoclusters. For instance, the fusion of the C390 module with a designed CTPR metal-coordination domain, allowed the stabilization of gold nanoclusters (C390-AuNC). The labelling of the protein with gold nanoclusters allowed the *ex vivo* correlation of the accumulation of C390-AuNC with the observed antifibrotic therapeutic effect [[26], [27], [28]].

These results confirmed that the C390 module drives the anti-fibrotic effect, highlighting the potential of CTPR-based therapeutics. Besides, the presence of AuNC helped understanding the molecular mechanism of the antifibrotic effect and thus can be used for drug development purposes. Building on this concept, here we propose the investigation of C390-AuNC as potential Hsp90 inhibitor in two chemically induced liver pathologies: carbon tetrachloride (CCl_4_) induced acute liver fibrosis to evaluate the ability of nanoclusters to reverse fibrosis, and diethylnitrosamine (DEN) + CCl_4_ induced hepatocellular carcinoma (HCC) to evaluate its capacity to reduce tumor growth.

## 2 Results and discussion

### 2.1 Synthesis, characterization & stability of C390-AuNC

**Fig. 1:**
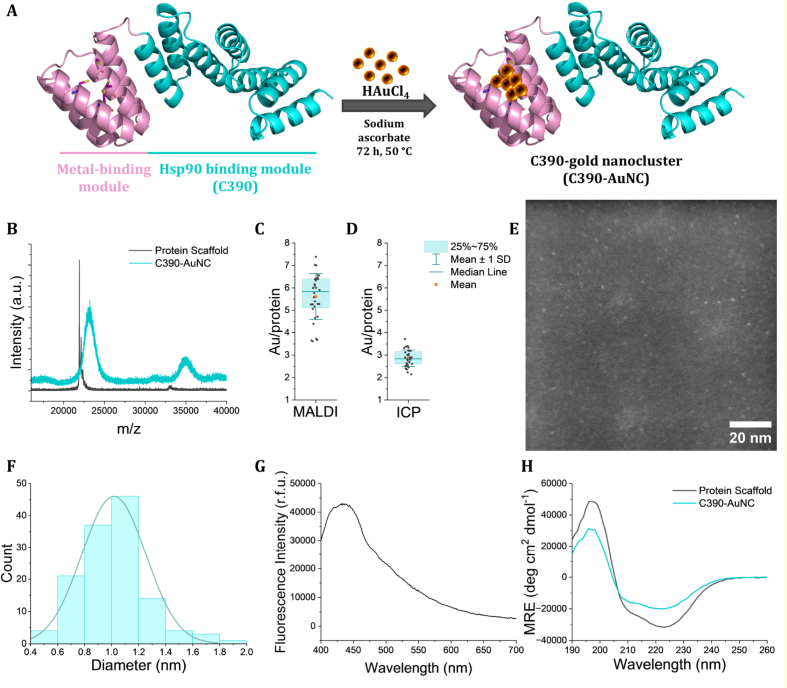
Synthesis and characterization of C390-AuNC. (A) Schematic representation of the chimeric protein and the synthesis of protein-stabilized gold nanoclusters (C390-AuNC). The protein structure was predicted using AlphaFold based on its sequence, in which four residues were mutated to cysteines (highlighted in magenta) to generate a metal-binding site. The C390 therapeutic module is shown in blue. (B) MALDI-TOF spectra of the protein scaffold (grey) and C390-AuNC (blue). The box plot shows the distribution of the number of gold atoms per protein, measured by (C) MALDI-TOF and (D) ICP-MS obtained from the different C390-AuNC batches synthesized and used throughout the study (n = 32). (E) HAADF-STEM image of the C390-AuNC. The scale bar represents 20 nm. (F) Size distribution of C390-AuNC obtained by the measurement of 130 particles in three different STEM images. (G) Fluorescence emission spectra of C390-AuNC excited at 360 nm. (H) Circular dichroism spectra of the protein scaffold (grey) and C390-AuNC (blue). Spectra shown on (B), (G), and (H) are representative spectra of the respective samples.

Aiming to develop multifunctional protein-based nanomaterials capable of reducing liver fibrosis and combating hepatocellular carcinoma, we used a previously described chimeric protein that integrates a four-cysteine metal binding site and the therapeutic Hsp90-binding motif, C390 (Fig. 1A). For simplicity, we will refer to this chimera protein as C390 throughout the text. The C390 multifunctional protein was successfully expressed and purified as confirmed by SDS-PAGE and MALDI-TOF (Figure SI1A – SI1C). Circular Dichroism (CD) spectra (Figure SI1D) exhibited the characteristic α-helical signature of CTPR proteins, with double minima at 208 and 220 nm.

The gold nanoclusters (AuNC) were synthesized *in situ* on the C390 protein scaffold, yielding C390-stabilized nanoclusters (C390-AuNC). First, the protein scaffold was incubated with gold salt (HAuCl_4_), followed by reduction of gold ions by sodium ascorbate leading to the formation of AuNC. Considering the large scale of material needed for the pre-clinical experiments, several batches of C390-AuNC were prepared. The formation and coordination of AuNC to the protein scaffold was verified by MALDI-TOF (Fig. 1B), which revealed a broadening and shift of the C390-AuNC peak to higher mass-to-charge ratios. This peak corresponds to the mass distribution of the protein-gold nanocluster hybrids, from which, based on the gold atomic mass and the protein molecular weight, an estimated gold loading per protein was calculated for each batch and subsequently averaged, yielding a mean value of 5.6 ± 1.0 atoms of gold per protein (Fig. 1C). An independent and quantitative determination of the gold loading was obtained by calculating the ratio of the measured gold content by ICP-MS and protein concentration (BCA method), which yielded an average value of 2.9 ± 0.4 gold atoms per protein (Fig. 1D). The discrepancy between the two values likely reflects the non-quantitative nature of MALDI-TOF for heterogeneous protein–nanocluster assemblies, as evidenced by the peak broadening observed in the MALDI-TOF spectra, as well as inaccuracies in protein quantification.

After confirming the coordination of gold to the protein scaffold, C390-AuNC was further analyzed by HAADF-STEM (High Angle Annular Dark-Field Scanning Transmission Electron Microscopy, Fig. 1E), in which AuNC could be observed as bright structures in the dark background. The inorganic core of C390-AuNC was measured to have 1.0 ± 0.2 nm diameter (Fig. 1F), confirming the formation of gold nanoclusters. X-ray photoelectron spectroscopy (XPS) analysis of Au 4f region, based on peak deconvolution and integration of the Au 4f7/2 components (Figure SI2), indicated that the gold nanoclusters were constituted by approximately a 1:1 ratio of Au^0^ and Au^1+^ species. This composition is in line with previous reports of gold nanoclusters [26,29,30]. Besides the inorganic core size measured by HAADF-STEM, colloidal properties, such as the hydrodynamic diameter and zeta potential, play an important role in determining nanomaterial fate and behaviour *in vivo*. The hydrodynamic diameter distributions measured by Dynamic Light Scattering (DLS) were centred at 5.1 nm in Mili-Q water and 6.4 nm in PBS (Figure SI3A-B), consistent with the predicted protein size of around 5 nm [31]. The zeta potential was found to be nearly neutral (−3.4 mV).

One of the most well-known and exploited characteristics of atomic gold clusters is their fluorescent properties [32]. C390-AuNC exhibited a maximum fluorescence emission at 430 nm upon excitation at 360 nm (Fig. 1G). These blue-emission properties are consistent with those previously reported for protein-stabilized AuNC of similar size [32].

Given the crucial role of protein structure in determining both the stability of the protein–nanocluster hybrid and the ability of the C390 module to interact with its binding partner Hsp90, we evaluated the secondary structure of the protein and the C390-AuNC by circular dichroism (Fig. 1). The spectra indicated that the synthesis did not disrupt the secondary structure of the protein, as C390-AuNC maintains the characteristic double minima of the α-helical conformation.

**Fig. 2:**
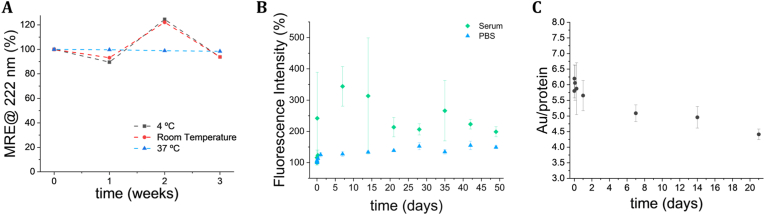
Stability of C390-AuNC under storage and physiologically relevant conditions. (A) Percentage of initial mean residue ellipticity (MRE) measured at 222 nm of the protein scaffold in C390-AuNC incubated at 4 °C, room temperature, or 37 °C for up to three weeks. (B) Fluorescence intensity at the emission maximum of the C390-AuNC incubated in PBS pH 7.4 and serum at 37 °C for over eight weeks. (C) Number of gold atoms per protein determined by MALDI-TOF after incubation in PBS pH 7.4 at 37 °C for up to 3 weeks. Data points on (B) and (C) correspond to the average of three replicates and the error bars to the respective standard deviation.

To assess the suitability of C390-AuNC for *in vivo* pre-clinical studies, we evaluated the colloidal and structural stability under both storage conditions (PBS pH 7.4, 4 °C or room temperature) and physiologically relevant conditions (PBS pH 7.4, 37 °C). For all the conditions tested the secondary structure of the protein scaffold remained unaltered, as verified by the superimposition of the CD spectra obtained for all the timepoints (Figure SI4A-C and Fig. 2A). The colloidal stability of the gold nanocluster component was also assessed by monitoring the variation of the fluorescence intensity along the time. C390-AuNC revealed similar stability under all the PBS-based conditions (Figure SI4D and Fig. 2B), with some increase in fluorescence intensity at 48 h that stabilized afterwards. This increase in fluorescence was accompanied by a decrease of approximately 1-1.5 gold atoms per protein (Fig. 2C). When incubated in serum, C390-AuNC showed a greater increase in fluorescence together with higher variability, particularly during the initial days of incubation, before gradually stabilizing and reaching values similar to the PBS conditions. The changes in fluorescence and gold content suggest potential minor alterations in the nanocluster composition rather than significant degradation. Importantly, the preservation of the secondary structure confirms the robustness of the C390 module over time. Overall, these results indicate that C390-AuNC exhibit structural, colloidal, and photophysical stability under all the relevant conditions.

### 2.2 *In vivo* animal studies in chemically-induced liver murine models

All animal studies were approved by local animal committee of UC Louvain, with approval number 2024/UCL/MD/002. All procedures were conducted in accordance with guidelines and relevant legislation from the Department of Animal Welfare, Brussels. Mice were housed up to five per cage randomly, kept under a 12- hour light/12- hour dark cycle in the animal facility. All mice were given free access to food and water unless otherwise indicated. C57BL/6 J and healthy C3H/HeNRj male mice were obtained from Janvier Laboratories (France). C3H/HeNRj mice with HCC were bred in Laboratory of Hepato-Gastroenterology, IREC, UCLouvain (Belgium).

#### 2.2.1 Efficacy study in liver fibrosis

**Fig. 3:**
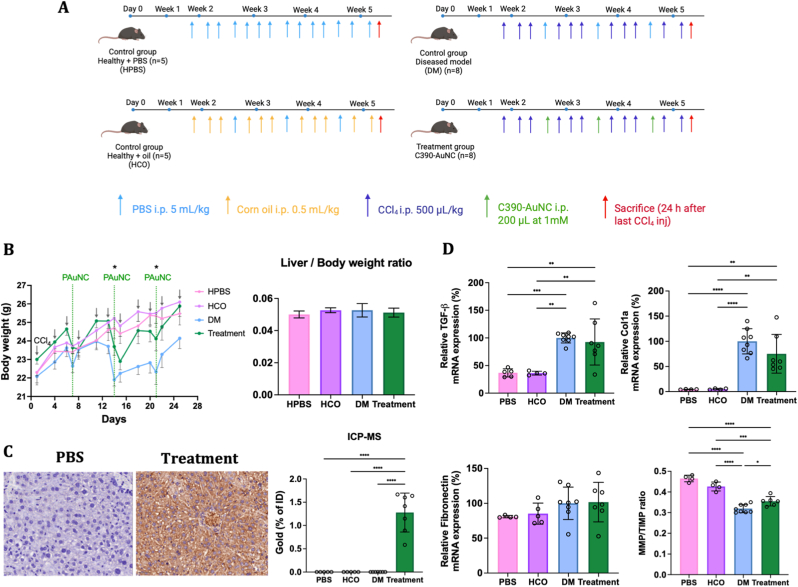
*In vivo* efficacy of C390-AuNC in liver fibrosis model. (A) Schematic illustration of CCl_4_-induced acute liver fibrosis model. The treatment mice were intraperitoneally administered CCl_4_ (500 μL/kg) and C390-AuNC (200 μL of 1 mM concentration). (B) Mice body weight and liver/body weight ratio. (C) IHC analysis to detect C390 protein, and ICP-MS to detect and quantify gold in liver tissues. (D) Relative mRNA expression of TGF-β, Col1a, fibronectin markers and MMP/TIMP ratio. Data points shown as mean ± SD, n = 5-8, Statistical analysis: One-way ANOVA followed by Tukey's post hoc test (∗p < 0.05, ∗∗p < 0.01, ∗∗∗p < 0.001, ∗∗∗∗p < 0.0001) (PBS: Healthy control, HCO: Healthy corn oil, DM: Disease control, Treatment: C390-AuNC treated mice).

To study the efficacy of C390-AuNC in reducing liver fibrosis, we performed an *in vivo* investigation in a CCl_4_-induced mice model as represented in Fig. 3A. CCl_4_ gets metabolized in the liver to produce reactive free radicals causing hepatocellular injury that activates a wound healing response. On repetitive CCl_4_ administration, the TGFβ1-dependant wound healing process slows down and causes accumulation of scar tissue and collagen in the liver [33]. Disease control group (DM) received PBS injections at the beginning of week 3, week 4 and week 5, while the treated group received C390-AuNC (Treatment). While CCl_4_ injections cause weight loss in all animals, the weight recovery was more rapid and complete in treated mice compared to disease control as seen in Fig. 3B. The body weight of the former was similar to that of healthy control mice at the end of the study (25.8 ± 0.6 g vs 26.1 ± 0.7 g, p > 0.05). The liver and body weight ratio showed no differences between all groups.

##### 2.2.1.1 C^39^0-AuNC quantification and safety evaluation

Adequate accumulation of protein in liver is required for impacting the fibrogenic process. Immunohistochemical (IHC) analysis of functional C390 showed equally dispersed protein in the liver tissue sections compared to healthy control which showed absence of C390 protein. C390-AuNC concentration in the liver was quantified using ICP-MS and corresponded to 1.3 ± 0.3% of the total injected dose (Fig. 3C). This result confirmed the retention of the protein inside liver tissues after 5 days post-treatment, thereby, facilitating the potential of C390-AuNC towards Hsp90 inhibition and suppression of TGF-β expression. Plasmatic transaminase levels (ALT/AST) were quantified at sacrifice (i.e. 24 h post last CCl_4_ injection) as markers of hepatic injury. No significant differences were observed between disease control and C390-AuNC-treated mice (Figure SI5A), indicating that the extent of CCl_4_-induced liver damage was comparable between groups.

##### 2.2.1.2 Fibrotic biomarker expression & thermal imaging

Scar tissue is composed of fibrillar collagen type 1 and fibronectin, and TGFβ1 is a main driver of the fibrotic process as described in Schematic 1A [34] [35,36]. The mRNA expression of ColΙα1, fibronectin and TGFβ1 in the liver was investigated by qPCR, exhibiting 8% and 25% reduction in relative expression of TGFβ1 and ColΙα1 genes in C390-AuNC-treated mice compared to disease control respectively, signifying less production of new collagen fibers (Fig. 3D). No significant differences were observed between any of the groups for fibronectin mRNA expression. MMP/TIMP ratio is commonly used to study degradation of collagen fibers in the liver, whereby, a high ratio indicates more fiber degradation [37]. Treated mice showed significantly higher MMP/TIMP ratio (individual graphs shown in Figure SI5B) than that observed for the diseased control (0.355 ± 0.02 vs 0.319 ± 0.03; p < 0.05). Decreased ColΙα1 production and higher degradation of collagen likely contribute to the reduction in liver fibrosis as seen in Fig. 3D.

Non-invasive real time thermal imaging (surface thermography) in diseased mice with inflammation can show an alteration in thermal energy emission [38]. Normal liver temperatures (∼37 °C) were observed for healthy controls PBS and HCO, whereas the diseased control and C390-AuNC treated mice showed temperature variances after 2 weeks of CCl_4_ injections (n = 5). However, treated mice showed less variances compared to diseased mice as shown inFigure SI5C. The heterogeneity in thermal profiles can be correlated to increased blood flow, morphological changes and increased immune cell infiltration in the liver. Thus, thermal variations could be used as an indicator for inflammation and disease progression in the liver.

##### 2.2.1.3 Histological & immunohistochemical analysis

**Fig. 4:**
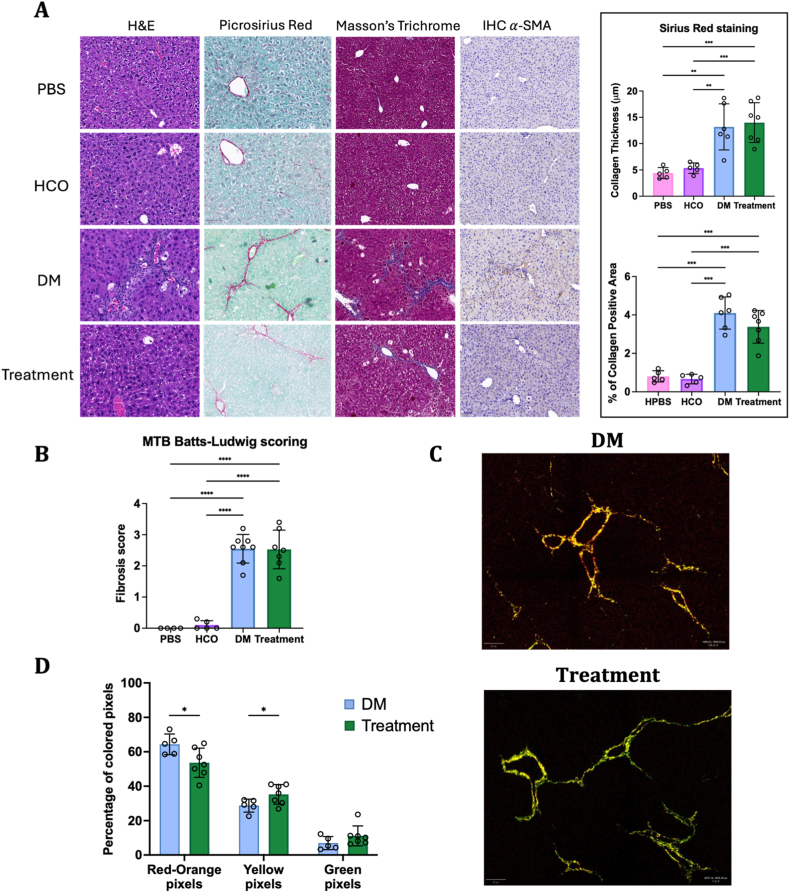
Histological analysis and PL microscopy of C390-AuNC in liver fibrosis model. (A) H&E, PSR, MTB staining and IHC of α-SMA performed in histological tissue sections of mice liver (Magnification 20x, scale bar – 100 μm). (B) Batts-Ludwig fibrosis scoring of MTB-stained tissue sections. (C) Differences in collagen fiber orientation observed using PL microscopy. (D) Percentage of red-orange, yellow- and green-colored pixels measured in PSR-stained sections with PL microscopy. Data points shown as mean ± SD, n = 5-8, Statistical analysis: One-way ANOVA followed by Tukey's post hoc test (∗p < 0.05, ∗∗p < 0.01, ∗∗∗p < 0.001, ∗∗∗∗p < 0.0001) (PBS: Healthy control, HCO: Healthy corn oil, DM: Disease control, Treatment: C390-AuNC treated mice).

Clinical investigation of liver fibrosis was performed by evaluating collagen deposition and histopathologic changes in tissue sections [39]. In mice, disease progression in liver is exhibited by infiltration of neutrophils during inflammation, evaluated by Hematoxylin and Eosin (H&E) staining, and generation of collagen fibers between blood vessels and capillaries during fibrosis, represented by red color staining collagen fibers in Picrosirius red staining (PSR) [40]. Moreover, Masson's trichrome blue (MTB) stain is used to stain collagen fibers in blue, and a standard scoring system - Batts-Ludwig scoring – which evaluates the topography of collagen bundles from initial thin strands (score 1) to thick hexagonal bridges (score 4) is used for quantification [39]. We performed H&E, PSR and MTB staining as seen in Fig. 4A. Compared to disease controls, C390-AuNC-treated animals showed less hepatocellular necrosis, less inflammation, and less collagen deposition (PSR & MTB), although, there was no significant difference in the Batts-Ludwig scoring (Fig. 4B), in the thickness of collagen bundles (Fig. 4A) or in the total amount of collagen in the liver (Fig. 4A).

Furthermore, IHC analysis of α-SMA in the diseased control shows greater deposition around the vessels compared to C390-AuNC treated livers, which is also compatible with less fibrogenesis.

Conventional histological analyses rely on qualitative or semi-quantitative methodologies that may lack sensitivity and accuracy for fibrotic changes. Polarization light (PL) microscopy of PSR-stained collagen fibers provides a better understanding and quantitative assessment of collagen fiber morphology and composition [41]. Collagen shows denser, cross-linked, high twisting and spindle fiber conformation during cirrhosis that illuminates as red- or orange-colored fibers under PL as seen in Fig. 4C for disease control. In contrast, green- or yellow-color shows loose and uncoiled collagen fibers for the C390-AuNC-treated mice. These pixels were measured as percentage of total pixels (Fig. 4D) and showed significant differences between disease control and treated mice (red-orange pixels: 64.3 ± 5% vs 53.6 ± 7%, p < 0.05 and yellow pixels: 28.7 ± 3% vs 35.9 ± 4%, p < 0.05). Collectively, this indicates that intraperitoneal administration of C390-AuNC nanohybrids reduces liver fibrosis in mice exposed to CCl_4_.

#### 2.2.2 Efficacy study in a murine hepatocellular carcinoma model

**Fig. 5:**
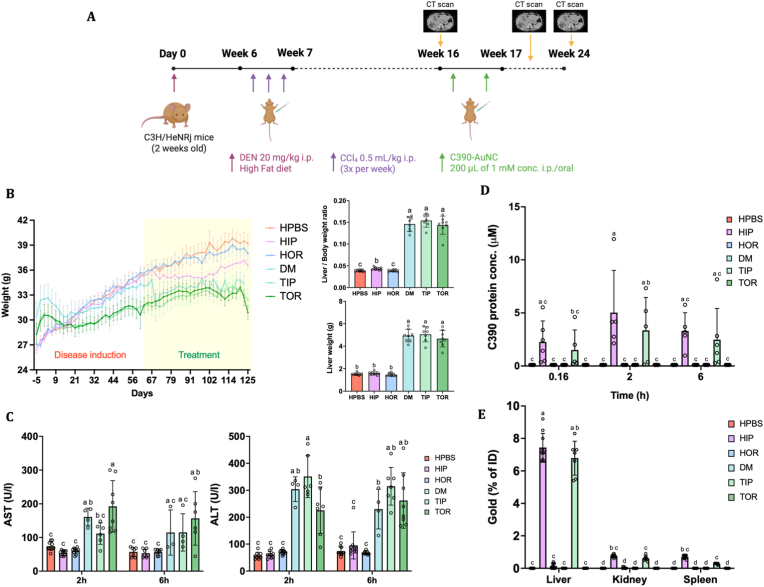
Pharmacokinetics, biodistribution and liver toxicity of C390-AuNC. (A) Schematic depicting the *in vivo* study plan in C3H/HeNRj mice using DEN + CCl_4_ combination for HCC induction and administration regimen of 2 doses of C390-AuNC treatment per week for eight weeks. (B) Body weight of mice plotted over time, liver/body weight ratio and liver weight plotted at the end of the study (n = 7-10, statistical analysis: One-way ANOVA followed by Tukey's post hoc test). (C) AST and ALT levels showing liver toxicity at 2 h and 6 h after first administration of C390-AuNC (n = 7-10, statistical analysis: One-way ANOVA followed by Dunnett's post hoc test). (D) Pharmacokinetic profile of C390-AuNC over time after first administration quantified using competitive ELISA (n = 5-10, statistical analysis: Two-way ANOVA followed by Tukey's multiple comparisons test). (E) Biodistribution study performed by ICP-MS to quantify gold conjugated to C390 in liver, kidney and spleen at the end of the study (n = 5 to 10, statistical analysis: Two-way ANOVA followed by Tukey's multiple comparisons test). (Data points shown as Mean ± SD; p < 0.05, same alphabet refers to non-significant differences between the groups). HPBS – healthy controls, HIP – healthy mice administered C390-AuNC i. p., HOR – healthy mice administered C390-AuNC orally, DM – diseased control with DEN + CCl_4_ induction, TIP – DEN + CCl_4_ mice administered C390-AuNC i. p., TOR - DEN + CCl_4_ mice administered C390-AuNC orally.

To further elucidate the potential of C390-AuNC in reducing hepatic cancer, we used a chemically induced hepatocellular carcinoma model (diethylnitrosamine injection 2 weeks after birth) in C3H/HeNRj male mice to induce genotoxicity and tumorigenicity, followed by repeated CCl_4_ injections (10 weeks) to cause injury and fibrosis. After tumor development, monitored by CT scan (see below), mice were treated with i. p. or oral administration of C390-AuNC twice per week, for eight weeks, as represented schematically in Fig. 5A. The groups were named as: HPBS – healthy controls, HIP – healthy mice administered C390-AuNC i. p., HOR – healthy mice administered C390-AuNC orally, DM – diseased control with DEN + CCl_4_ induction, TIP – DEN + CCl_4_ mice administered C390-AuNC i. p., TOR - DEN + CCl_4_ mice administered C390-AuNC orally.

In Fig. 5B, we measured the body weight of mice and plotted it against time during disease induction and treatment. We observed a steady increase in the body weight of healthy control mice. However, mice induced with HCC showed a slight increase of weight initially and then a saturation or plateauing in body weight after a week of disease induction. This saturation remained consistent even during treatment for both disease control (DM) and C390-AuNC treated mice (TIP & TOR). On comparing liver body weight ratio and liver weight of all groups (Fig. 4B), the healthy mice showed low weight compared to DEN-CCl_4_ induced mice due to inflammation and tumor generation in the latter. There were no statistical differences observed in liver weights between the healthy control (HPBS) and healthy mice treated with i. p. (HIP) or oral (HOR) formulations of C390-AuNC, proving lack of any adverse effects caused due to the nanoclusters (HPBS: 1.51 ± 0.06 g vs HIP: 1.58 ± 0.07 g vs HOR: 1.46 ± 0.08 g, p > 0.05).

##### 2.2.2.1 Liver toxicity

For liver toxicity, AST and ALT levels were measured in the plasma 2 h and 6 h post first injection of C390-AuNC. As seen in Fig. 5C, the healthy controls receiving the protein treatment did not show any increase in AST/ALT levels, compared to healthy control receiving no treatment, thereby validating lack of side effects or no liver toxicity caused by C390-AuNC. However, in contrast, the disease control (DM) and diseased mice treated with orally administered C390-AuNC (TOR) showed a significant increase in AST levels at 2 h compared to non-significant differences observed between healthy control and i. p. treated mice (HPBS vs TIP, p < 0.05 (HPBS: 74 ± 11U/L vs TIP: 111.3 ± 30 U/L, p > 0.05). Thus, the protein nanohybrid C390-AuNC does not cause any liver toxicity by itself but in diseased conditions it helps to reduce AST levels comparable to healthy mice.

##### 2.2.2.2 Pharmacokinetic and biodistribution study

For the pharmacokinetic study, blood was collected at 10 min, 2 h and 6 h post-injection or oral gavage of the first dose of treatment with C390-AuNC. Using competitive ELISA for quantification of the C390 protein present in the C390-AuNC, the highest C390 accumulation was detected at 2 h after injection for the healthy mice treated intraperitoneally (HIP: 5.03 ± 3.9 μM) and diseased mice treated intraperitoneally (TIP: 3.34 ± 3.1 μM). We observed that the i. p. treatment showed approximately 5 μM concentration of protein in the blood, whereas there were no protein traces observed in the oral administration group (Fig. 5D). This was confirmed by the biodistribution study using ICP-MS, whereby after two months of treatment when the mice were sacrificed, approximately 7.5% of the total injected dose was observed in HCC induced mice given i.p. doses of C390-AuNC as seen in Fig. 5E. However, no protein was observed in the liver for the orally administered group. Furthermore, low concentration (<1%) of protein-gold nanoclusters was observed in the kidney and spleen showing high liver targeting for i. p. administered C390-AuNC nanoclusters. Since no protein reached the liver for the oral administration group, it was excluded in further studies. Biodistribution was assessed at the endpoint and therefore reflects the residual fraction of the administered dose, with most of the material being expected to have been cleared at earlier timepoints. Nonetheless, the low concentrations of gold detected in the kidney and spleen are consistent with efficient renal filtration and urinary excretion, typically described for ultrasmall gold nanoclusters such as those described here, with a core of 1 nm and a hydrodynamic diameter of less than 6 nm [[42], [43], [44]].

##### 2.2.2.3 Tumor growth and thermal analysis

**Fig. 6:**
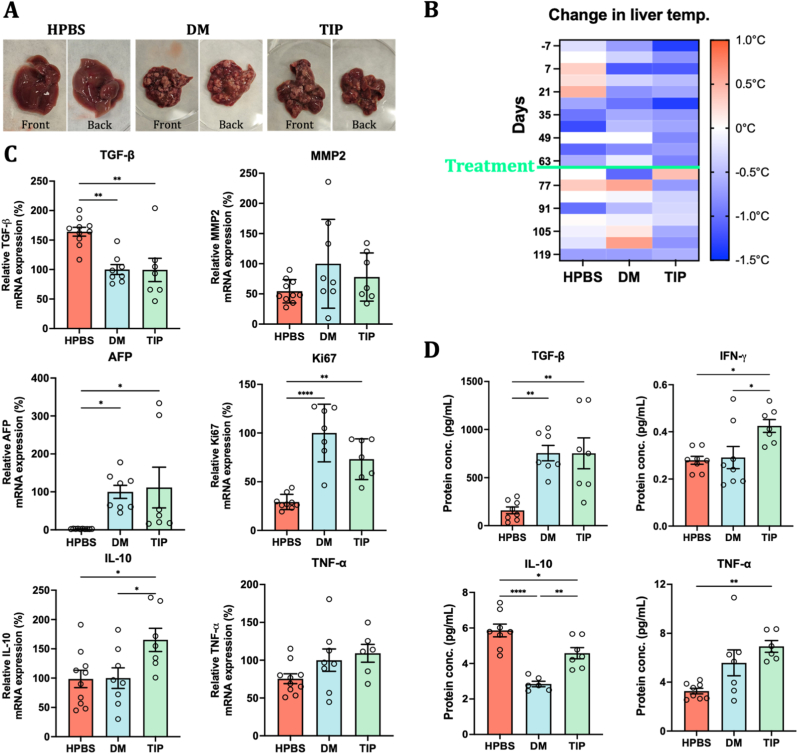
Anti-tumor efficacy of C390-AuNC in the DEN + CCl_4-_induced HCC mouse model. (A) Representative macroscopic qualitative images of livers showing significantly higher tumor growth in diseased control compared to i. p. C390-AuNC treated livers. (B) Real-time thermal variations measured in the liver before and after treatment (n = 5). (C) Relative mRNA expression of fibrotic markers (TGF-β and MMP-2), cancer markers (AFP and Ki67) and inflammatory markers (IL-10 and TNF-α) measured using qPCR (n = 7-10, statistical analysis: One-way ANOVA followed by Tukey's post hoc test). (D) Protein expression of TGF-β and IFN-γ, IL-10 and TNF-α cytokines measured by ELISA (n = 7-10, statistical analysis: One-way ANOVA followed by Tukey's post hoc test). (Data points shown as Mean ± SD; p < 0.05, same alphabet refers to non-significant differences between the groups).

After sacrificing the mice, we collected the livers to observe tumor growth macroscopically. Images in Fig. 6A highlight significant tumor growth with fleshy, tan appearance of tumors on the anterior and posterior views of the liver of disease control mice. In contrast, i. p. C390-AuNC-treated mice showed substantially reduced tumor growth indicating a tumor regression under treatment with C390-AuNC. Furthermore, micro-CT images of livers from disease control mice showed larger number of tumors with low density (indicating intra tumor necrosis) whereas C390-AuNC treated livers contained small number of tumors with a solid core as seen in **Video**.

Real-time thermal imaging of the livers showed uniform thermal homeostasis in livers of healthy mice compared to an increase in liver temperatures of disease control and C390-AuNC treated mice during DEN + CCl_4_ induction, as expected (Fig. 6B). However, once the treatment was initiated, C390-AuNC group showed a slight reduction and stabilization of thermal energy emission (±0.1 °C) as opposed to further increase in emissions observed for the diseased control group (±0.5 °C). This trend is a potential indicator of better immune response and recovery in C390-AuNC treated mice.

##### 2.2.2.4 mRNA and protein expression

Common cancer and inflammatory biomarkers were quantified by qPCR for mRNA expression and ELISA for protein expression, to investigate the efficacy of C390-AuNC in suppressing tumor growth and modulating an immune response. Liver samples containing tumor & non-tumor tissues were used. Although no changes in mRNA expression of TGF-β between disease control vs C390-AuNC were observed (Fig. 6C), a 22% reduction was noted for MMP-2 marker which is overexpressed in HCC and may represent a pro-invasive/pro-metastatic cue (**schematic 1B**). Clinically used cancer markers like Ki-67 (a marker for cell proliferation) and α-fetoprotein (AFP) (expressed during fetal development and re-expressed in HCC) are used to study tumor growth and its aggressiveness. The mRNA expression of Ki-67 was suppressed by 27% (though not statistically significant) whereas AFP expression was reduced in 5 out of 7C^39^0-AuNC treated mice. Furthermore, a strong anti-inflammatory response was observed in the treatment group as seen in Fig. 6C with a significant increase of IL-10 mRNA expression. This was confirmed by 66% increase in IL-10 protein expression in liver samples of the C390-AuNC-treated group (Fig. 6D). The expression of TNF-α, whether mRNA or protein, was not significantly affected by the treatment. Additionally, cytokine IFN-γ also increased by 49% in tumors of C390-AuNC-treated mice, which suggests induction of apoptosis of cancer cells [45]. Other cancer and inflammatory markers were also measured to study the effects on cross-talk between oncogenes and oncoproteins (Figure SI6&7).

This decrease in MMP-2, Ki-67, and AFP expression levels with the concomitant increase in IL-10 and IFN-γ supports the qualitative observation that the C390-AuNC treatment has an anti-tumor activity in the HCC model under study at the molecular level.

##### 2.2.2.5 Histological and immunohistochemical analysis

**Fig. 7:**
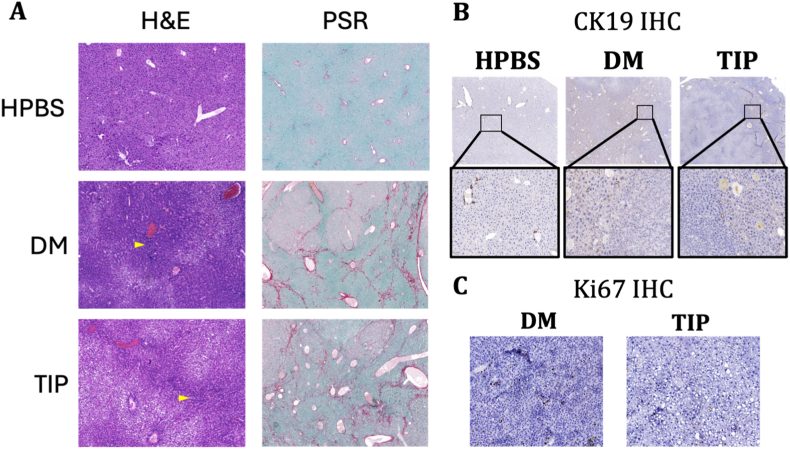
Histological and Immunohistochemical analysis of tumor growth in HCC mouse model. (A) H&E, PSR and MTB staining on whole liver sections containing tumor & non-tumor tissue. (B&C) Immunohistochemical detection of CK-19 and Ki-67 proteins in liver tissue sections containing tumor and non-tumor cells (Magnification 20x, scale bar – 100 μm).

We performed further investigation using histopathological and IHC analysis as shown in Fig. 7A, B & C. The liver sections showed high bile duct proliferation in H&E-stained tissues of DEN + CCl_4_ injected mice, while PSR showed high intensity of collagen fibres around the tumors. IHC analysis of cytokeratin-19 (CK-19) unveiled the presence of bile ducts or ductular proliferation in the periphery of the nodules and absence of portal triad inside the nodules confirming the cancerous nature of the tumors. Ki67 further confirmed high cell proliferation inside the cancerous nodules with lower cell proliferation in C390-AuNC-treated liver tumors. A high concentration of protein-stain (brown color) around the tumors in diseased control liver section compared to a light intensity of protein surrounding tumors in C390-AuNC-treated mice showed less cell proliferation and slow tumor growth in treated mice. This histological and immunohistochemical data assessment further confirm the antitumoral capacity of C390-AuNC.

## 3 Conclusion

An engineered C390 protein mimicking naturally occurring Hsp90 recognizing domains provides great potential for targeting fibrosis and cancer by inhibiting the release of TGF-β that is dependent on Hsp90. This scaffold can be engineered to display metal binding domains which can stably coordinate metals like gold or gadolinium, thereby, providing imaging/diagnostic ability to the therapeutic nanohybrid system.

We investigated the capability of a protein-nanocluster hybrid formulation (C390-AuNC) to reduce fibrosis and tumor growth in pre-clinical murine models. The data collected from the efficacy studies collectively suggest that C390-AuNC act as anti-fibrotic agent. In the acute fibrotic model, this was highlighted by the reduction in the expression of fibrotic markers like Collα1 gene by 25% and the increase in degradation of collagen fibers by 11% measured as increase of MMP/TIMP ratio in C390-AuNC treated livers. This was further confirmed by polarization microscopy, showcasing loose collagen strands and lack of bridging in liver sections of C390-AuNC-treated group *versus* the thick spindle fibres forming hexagonal bridges in disease control. In the chronic HCC model, 2 doses per week of C390-AuNC showed noteworthy reduction in tumor burden with an increase in mRNA and protein expression of cytokines IL-10 (∼65%) and TNF-α (24%). Furthermore, AFP expression (a fetal protein re-expressed by cancer cells) and cell proliferation in tumors were reduced by C390-AuNC treatment. Macroscopic images and microCT scans also showed smaller number of tumors in C390-AuNC-treated mice.

Altogether, C390-AuNC hybrids show great potential as a future clinical anti-fibrotic and anti-cancer agent by potentially inhibiting Hsp90 protein and blocking TGF-β pathway. Furthermore, they could be used as part of a combination therapy by conjugation with Rapamycin, an immunosuppresant that shows anti-cancer activity, thereby reducing immunosuppression after liver transplant, and C390 accumulated in the tumor expressing anti-cancer activity, preventing relapse of cancer cells. Additionally, this study paves the way for the development of engineered protein-based theranostics, potentially improving treatment and patient outcomes.

Analogously, to what has been shown for cardiac fibrosis, this study demonstrates that CTPR proteins and in particular the C390 therapeutic module is amenable to protein engineering and allows the introduction of a metal stabilizing module and the effective stabilization of metal nanoclusters without impairing its therapeutic activity or imparting toxicity. Importantly, their modularity opens the possibility to tailor the metal component to the desired imaging modality. In the specific case of liver fibrosis and HCC, for example, gold nanoclusters could be substituted by iron-based nanostructures to allow treatment follow up in real time by magnetic resonance imaging (MRI), one of the most widely used techniques in the diagnosis of these diseases, without the need for the administration of an additional contrast agent. This study provides evidence not only on the promise of C390 as an antifibrotic and antitumoral agent but also on its versatility to be further engineered as a theranostic agent with the potential of improving treatment and patient outcomes.

## 4 Materials and methods

### 4.1 Protein design, expression and purification

The consensus tetratricopeptide repeat (CTPR) protein scaffold used in this study was previously designed and characterized [26]. In brief, the metal binding module was previously engineered based on the CTPR concave surface (PDB ID: 2HYZ): two CTPR units were repeated in tandem and the residues 5 and 9 of the first CTPR unit and residues 2 and 6 of the second unit were substituted with cysteines, thereby generating a metal binding motif. This metal binding module was then fused to the N terminal of the C390 module [24], originating a chimeric multifunctional protein. DNA sequencing (Stab Vida) was used to verify the DNA sequence of the gene:

GAMGSAEAWCNLGCAYYKQGDYDEAIEYYQKALELDPRSACAWYCLGNAYYKQGDYDEAIEYYQKALELDPRSAEAWKNLGNAYYKQGDYQKAIEYYQKALELDPNNASAWYNLGNAYYKQGDYQKAIEYYQKALELDPNNAKAWYRRGNAYYKQGDYQKAIEDYQKALGLDPNNAKAKQNLGNAKQKQG.

The gene coding for the designed protein was cloned into the pProEx-HTA vector, containing the sequence for an N-terminal hexa-histidine tag and for ampicillin resistance. The plasmid containing the gene for the protein of interest was transformed into C41 (DE3) cells, and overnight cultures were prepared by inoculation of LB medium containing 0.1 mg/mL ampicillin with a single colony of the transformed cells. 1 L cultures were set-up by diluting the overnight culture 1:100 in fresh LB medium with 0.1 mg/mL ampicillin and grown at 37 °C and 220 rpm until reaching an optical density between 0.6 and 0.8. At this point, 1 mM isopropyl β-d-thiogalactoside (IPTG) was added to induce protein expression, and the cultures were incubated for further 18 h at 20 °C. The cells were then centrifuged at 4500 rpm for 15 min and the resulting pellet was resuspended in lysis buffer (50 mM Tris, 500 mM NaCl, pH 8.0) and frozen at −20 °C. Afterwards, pellets were sonicated, centrifuged at 10000 rpm for 45 min, and the soluble protein fraction was purified by affinity chromatography using a 5 mL HisTrap Q column (GE Healthcare). After adsorbed to the column, the proteins were washed with a buffer containing 1 M NaCl, and subsequently eluted using 50 mM Tris, 500 mM NaCl, 300 mM imidazole, pH 8.0. The hexa-histidine tag was cleaved by incubation with Tobacco Etch Virus (TEV) protease for 18 h at room temperature. Both TEV and hexa-histidine tag were removed from the protein solution by affinity chromatography. The protein concentration was determined through the Beer-Lambert law, measuring the solution absorbance at 280 nm and using the extinction coefficient determined from the protein sequence (Expasy ProtParam).

#### 4.1.1 Synthesis of C390-AuNC

The protein-stabilized AuNC (C390-AuNC) were synthesized by scaling-up a method previously described by our group [26]. The purified protein scaffold was dialyzed to 50 mM PB, 150 mM NaCl, pH 10 for at least one day, changing the dialysis buffer thrice. 400 mL of the protein solution at 20 μM were transferred to a 1-L round bottom flask and incubated with 2.4 mL of 0.1 M HAuCl_4_ (30 eq. in relation to the protein) for 1 h at 25 °C. After this step, 40 mL of 1 M sodium ascorbate (3000 eq. respect to protein) were added to the reaction mixture and incubated for 72 h at 50 °C. The gold nanoparticles were separated from the C390-AuNC by centrifuging the mixture at least three times for 1 h at 17000 rpm. The supernatant was then concentrated to around 3 mL using a Pellicon® XL 50 Ultrafiltration Cassette (NMWCO 10 kDa). The C390-AuNC were then further purified and buffer exchanged to PBS pH 7.4 by gel filtration using a Sephadex™ G-25 resin (PD-10 Column, cytiva). C390-AuNC were concentrated to 1 mM of protein using Amicon® Ultra Centrifugal Filter (10 kDa MWCO) and the samples frozen with liquid nitrogen and kept at −80 °C until further use.

#### 4.1.2 Matrix assisted laser desorption ionization (MALDI) mass spectrometry

Samples were buffer exchanged to water and diluted to a protein concentration between 10 and 20 μM. 1 μL of each sample was then mixed with 3 μL of sinapinic acid (4-Hydroxy-3,5-dimethoxycinnamic acid, 10 mg/mL in 50:50 water/acetonitrile with 0.01% TFA) directly in the MALDI target and allowed to air dry. Mass spectra were acquired using a MALDI/TOF-TOF MS neofleX (Bruker) on positive reflection mode.

#### 4.1.3 Inductively coupled plasma mass spectrometry (ICP-MS)

Firstly, the C390-AuNC samples were diluted 50 times with water. Then, the gold in C390-AuNC was digested by placing 40 μL of diluted sample in 400 μL aqua regia freshly prepared. The Au concentration was then determined by measuring the samples using an iCAP-Q ICP-MS (Thermo Scientific, Bremen, Germany) equipped with an autosampler ASX-520 (Cetac Technologies Inc., NE, USA) (n = 3) and QtegraTM v2.6 (Thermo Scientific).

#### 4.1.4 High Angle Annular Dark-Field Scanning Transmission Electron Microscopy (HAADF-STEM)

The HAADF-STEM sample was prepared by drop-casting 0.5 μL of C390-AuNC at 15 μM, dispersed in miliQ-water onto a previously O_2_/UV cleaned Lacey 400 mesh Copper grid (TED PELLA INC., 01824). After 1 min, the grid was washed by touching parafilm with a drop of miliQ-water at an 90° angle. The grid was then allowed to fully dry before being imaged. HAADF-STEM images were obtained using a JEOL JEM-2100 F UHR microscope operated at 200 kV in scanning mode. The probe size was 1.5 nm and the inner detector angle was 75 mrad (HAADF).

#### 4.1.5 Circular dichroism (CD)

Circular dichroism spectra were acquired using a JASCO J-815 CD spectrometer (JASCO Corporation, Tokyo, Japan) in a quartz cuvette with 1 mm path-length. The spectra were obtained as an average of three accumulations. The samples were measured at a protein concentration of 10 μM.

#### 4.1.6 X-ray photoelectron spectroscopy (XPS)

The X-ray photoelectron spectroscopy spectrum was acquired in a Versaprobe III Physical Electronics (ULVAC) spectrometer with a monochromatic X ray source (Aluminium Kα line of 1487 eV), calibrated using the 3d5/2 line of Ag at 368.26 eV, with a spot-size of 100 μm, 0.05 eV of step energy, 55 eV of pass energy, and 50 ms per step. C390-AuNC dispersed in water was deposited by drop casting on a non-conductive tape and mounted on the sample holder once dried. Before sample measurement, Z-alignment was performed for optimal sample height. Calibration was done based on Carbon 1s C-C sp3, fixed at 284.8 eV and the spectrum analyzed using CasaXPS (2.3.16 PR 1.6), and the peaks assigned in agreement with ref [46]. To determine the oxidation states of C390-AuNC, the Au 4f7/2 peak was deconvoluted, and the proportion of Au^1+^ to Au^0^ was determined based on the ratio between the areas under the two corresponding peaks, centred at 84.7 and 83.7 eV.

#### 4.1.7 Dynamic Light Scattering (DLS)

The hydrodynamic diameter and zeta potential distributions of C390-AuNC were measured by DLS using a Zetasizer Ultra Red (Malvern). The hydrodynamic diameters were measured with the sample dispersed in PBS pH 7.4 or Mili-Q water, while the zeta potential was measured in water.

#### 4.1.8 Fluorescence measurements

50 μL of each sample at a protein concentration of 100 μM was placed in a black 384 well microplate (Corning) and the fluorescence spectra were acquired in a Synergy H1 Hybrid Multi-Mode Microplate Reader controlled by Gen5 Software. For the spectra measurements, the samples were excited at 360 nm, and the emission was recorded from 400 to 700 nm, with a step size of 1 nm. To be able to better compare the fluorescence values among samples, endpoint measurements were also acquired. In this case, the excitation was set to 360 nm and the emission to 430 nm.

### 4.2 *In vivo* animal studies in chemically induced liver murine models

#### 4.2.1 Acute liver fibrosis model

Seven-week-old male C57BL/6 J mice were kept on a normal diet (ND, SAFE Diets A03) and divided into 4 groups – Healthy control (HPBS, n = 5), Healthy corn oil (HCO, n = 5), disease control (DM, n = 8) and protein-gold nanocluster (C390-AuNC) treatment (Treatment, n = 8).

Liver fibrosis was chemically induced in DM and treatment groups by injecting 3 doses/week of 500 μL/kg body weight of carbon tetrachloride (CCl_4_) intraperitoneally (i.p.) in corn oil medium as seen in Fig. 3A. Since CCl_4_ induced fibrosis is reversible, it produces an acute condition and requires regular injections to maintain the fibrotic stage in the liver. HPBS and HCO groups were injected with 3 i. p. Doses/week of 5 mL/kg and 500 μL/kg body weight of PBS and corn oil respectively. After one week of induction, one dose/week of 200 μL of C390-AuNC at 1 mM (concentration of protein, determined by BCA assay) was injected i. p. Between the CCl_4_ injections for 3 weeks. The dose was determined based on the highest protein concentration that could be achieved while maintaining the colloidal stability. Mice were sacrificed 24 h after the last CCl_4_ injection.

The body weight of mice was recorded before the CCl_4_ injections (4 times per week) and the liver weight was measured after sacrifice to compare the liver/body weight ratio.

##### 4.2.1.1 C^39^0-AuNC quantification and safety evaluation

The localization of C390 in the liver tissue was studied by IHC analysis using polyclonal rabbit *anti*-C390 antibody (1:100, antibody platform from Unidad de Ensayos Biotecnológicos y Biomédicos, Servicios Científico Técnicos, Universidad de Oviedo, Spain) and Envision anti-rabbit HRP secondary antibody (Dako K4003), followed by diaminobenzidine (Dako K3468) to reveal the peroxidase activity (brown coloring), and the sections were counterstained with hematoxylin. The images were analyzed using QuPath software [47].

The accumulation in liver was measured by quantifying the amount of gold conjugated to the protein using inductively coupled plasma mass spectrometer (ICP-MS) (quadrupole ICP-MS: Thermo Fisher Scientific iCAP Q ICP-MS) [26]. The instrument was calibrated with 5% HCl as blank and Merck Certipur ICP gold standards were prepared at 1, 2, 5, 10, 20, 50 and 100 ppb gold. To quantify the gold in liver, samples were weighed and suspended in 2 mL of aqua regia solution (HNO_3_/HCl = 1:4) and digested in Precellys® Evolution Touch Tissue Homogenizer (France) with 3 cycles of 6000 rpm for 10 s each. Once the chlorine evaporated, 2 mL of H_2_O_2_ was added until the solution became clear and transparent. The solution was cooled and diluted to 10 mL with 5% HCl and subsequently analyzed with ICP-MS.

The liver damage was measured by quantifying plasmatic transaminase levels (ALT/AST) using a Fujifilm DRI-CHEM NX500 (Belgium) [48]. Blood samples were collected after sacrifice in the liver fibrosis study and at 2 h and 6 h after first C390-AuNC treatment during the HCC study. Plasma was extracted from the blood by centrifugation (4000 rpm, 15 min, 4 °C) and 10 μL was loaded into chips for quantifying ALT and AST levels.

##### 4.2.1.2 Fibrotic biomarker expression & thermal imaging

The relative mRNA expression of common fibrotic markers was measured using quantitative polymerase chain reaction (qPCR). RNA was extracted from liver tissue using TRIzol (Invitrogen, Thermo Fisher Scientific, Belgium). cDNA was synthesized from 1 μg of RNA and the gene expression was evaluated as per previously described (Q-Rex, Qiagen, Hilden, Germany) [49]. mRNA levels are normalized to ribosomal protein L19 (RPL19) as a reference gene. The results were expressed using “delta-delta Ct” method as percentage of the control group [50]. The genes analyzed are listed as follows: (A) For acute liver fibrosis study - *TGFβ*, transforming growth factor beta; *Col1a*, collagen type 1; *FN*, fibronectin; *MMP*, matrix metalloproteinases; *TIMP*, tissue inhibitor of metalloproteinases; (B) For HCC study, in addition to genes mentioned above - *Il-6*, interleukin 6; *IL-10*, interleukin 10; *IL-12*, interleukin 12; *Tnfα,* tumor necrosis factor alpha; *Hsp90aa*, heat shock protein aa; *Hsp90ab*, heat shock protein ab; *H19; IGF2,*insulin-like growth factor 2; *Ki67,* kiel 67; *AFP,* alpha fetal protein; *CK19,* cytokeratin 19.

Real-time infrared thermal imaging (IRT) in mice was performed twice per week using FLIR One Gen 3 thermal camera to study the inflammation in liver due to disease induction [51,52]. Mice were anesthetized under isoflurane (1.5-2%) and placed on their backs. A stage was placed above the mice to maintain a constant distance between the mice and camera. The camera was placed on the stage to record thermal & visual images of the thoracic region. The images were analyzed using Thermal studio suite software. The skin temperature was removed using background cancellation and the temperature detection range was set between 30 and 37 °C. An average of 5 data points was recorded per image and tabulated as a heat map on GraphPad Prism. Normal liver temperatures (∼37 °C) were observed for healthy controls HPBS and HCO, whereas the diseased control and C390-AuNC treated mice showed temperature variances after 2 weeks of CCl_4_ injections. However, treated mice showed better homeostasis compared to diseased mice as shown in Figure SI5C. Similar methodology was followed for the HCC study.

##### 4.2.1.3 Histological and immunohistochemical analysis

After sacrifice, liver sections were submerged in 4% paraformaldehyde (PFA) and fixed in paraffin for histological analysis. The fixed liver sections were stained with hematoxylin and eosin (H&E), Picrosirius red (PSR), or Masson's trichrome blue (MTB) stain for both fibrosis and HCC *in vivo* studies. The stained sections were visualized under a brightfield microscopy to study neutrophil activation and bile duct proliferation (H&E), collagen fiber generation (SR) and severity of collagen deposition (MTB). The intensity of picrosirius red and thickness of collagen fibers were quantified on QuPath software and plotted as percentage of collagen positive area and collagen thickness (μm) respectively. The fibrosis score was calculated using Batts-Ludwig standard scoring system to study extent of hepatitis, fibrosis and cirrhosis in the liver fibrosis study [39].

Paraffin-fixed liver sections (4 μm) were used for immunohistochemical detection of α-SMA (alpha-smooth muscle actin), Ki-67 or cytokeratin-19 (CK-19). Briefly, a, polyclonal rabbit anti-mouse α-SMA antibody (1:200, Abcam, ab5694), monoclonal rabbit ki-67 antibody (1:400, Cell signaling, 12202), monoclonal rat CK-19 antibody (1:400, DSHB) and Envision anti-rabbit HRP secondary antibody (Dako K4003) were used, followed by diaminobenzidine (Dako K3468)) to reveal the peroxidase activity (brown coloring), and the sections were counterstained with hematoxylin.

PSR-stained liver sections were visualized under a PL microscope and the red-orange, yellow and green pixels are quantified using a QuPath script [41] based on RGB threshold values. These values are plotted as percentage of all colored pixels in designated tissue area (n = 5-7).

#### 4.2.2 Murine hepatocellular carcinoma model

In theFig. 5A, C3H/HeNRj mice strain were injected with DEN 20 mg/kg intraperitoneally after two weeks of birth. The model is commonly used to induce hepatocellular carcinoma in liver murine model. After six weeks, the mice were injected with carbon tetrachloride thrice per week intraperitoneally at 0.5 mL/kg of body weight. During this entire period, the mice were also given a high fat diet, 60 kcal% fat (HFD, D12492 ResearchDiets, USA) to increase lipid metabolism in the liver. After 16 weeks, the mice were switched to a normal diet and the CCl_4_ injections were stopped. The mice were injected with 200 μL of C390-AuNC twice per week, i. p. or orally at a protein concentration of 1 mM for eight weeks. MicroCT scans were performed on day 1, day 28 and day 56 after starting treatment, to study tumor growth in the mice liver using contrast agent ExiTron® 12,000. After 24 weeks of *in vivo* study, the mice were sacrificed, and the livers were harvested.

##### 4.2.2.1 Pharmacokinetics studies

The levels of protein scaffold in mouse serum at different timepoints post administration were quantified by a competitive ELISA using an antibody specific to the C390 module. The *anti*-C390 antibody was purified from rabbits immunized with the C390 protein module, produced through the antibody platform of the Unidad de Ensayos Biotecnológicos y Biomédicos, Servicios Científico Técnicos, Universidad de Oviedo, Spain.

For the assay, Nunc MaxiSorp 96 well plate (Thermo Scientific™) was coated by incubation with 50 μL per well of the *anti*-C390 antibody (1:200 dilution) for 18 h at 4 °C. Afterwards, the antibodies were blocked with casein blocking buffer (Sigma C.7594) for 2 h at room temperature. A competitive reaction was then performed by loading 50 μL of each sample or calibration standard together with 50 μL of HRP-labelled protein scaffold (1:25000 dilution). The HRP-labelled protein scaffold was prepared using the same protein used for nanocluster synthesis (100 μL at 45.6 μM in PBS) using HRP Conjugation Kit - Lightning-Link® (Abcam, ab102890) following the manufacturer's instructions and stored at 4 °C until use. Following 1 h incubation at room temperature, plates were washed three times with PBS-T (0.1% Tween-20). The activity of bound HRP, that is inversely related to the concentration of the protein scaffold in the sample, was detected by adding 100 μL of 1-Step Ultra TMB ELISA (Thermo Scientific), producing a colorimetric signal. After 1 min and 20 s, the reaction was stopped with 50 μL sulfuric acid at 1 M. The absorbance at 450 nm was then measured to determine the concentration of the C390 protein scaffold in each serum sample and calibration standard. Protein scaffold concentrations in serum samples were calculated from standard curves generated for each plate using C390-AuNC as standards (20–2.5 nM protein) diluted in mouse serum to match the sample matrix. Serial sample dilutions from 1:50 to 1:32,000 were analyzed.

##### 4.2.2.2 Biodistribution study

After sacrificing the mice, the distribution of C390-AuNC in different organs was quantified by measuring accumulation of gold in the liver, spleen, and kidneys by ICP-MS, similarly to the method stated in section 4.2.1.1 except for number of cycles during homogenization. Samples were weighed and suspended in 2 mL of aqua regia solution (HNO_3_/HCl = 1:4) and digested in Precellys® Evolution Touch Tissue Homogenizer (France) with 3 cycles for liver and 6 cycles for kidneys and spleen, of 6000 rpm for 10 s each. Once the chlorine evaporated, 2 mL of H_2_O_2_ was added until the solution became clear and transparent. The solution was cooled and diluted to 10 mL with 5% HCl and subsequently analyzed with ICP-MS.

##### 4.2.2.3 Tumor growth - liver images and microCT

Whole livers were harvested at the end of the animal study and images of the anterior (front) and posterior (back) sides of the liver were photographed with an Android camera. Qualitative assessment of tumor number and volume was performed based on these images. For the microCT analysis of tumor growth over the duration of treatment phase, mice had to be transported to another facility which limited injecting C390-AuNC in treated mice. Thus, only diseased control mice (n = 4) were scanned for tumors throughout the treatment phase while the i. p. Treated mice (n = 1) were scanned on the day of sacrifice. This was due to ethical constraints of transporting mice back and forth between different facilities. This data was only used for qualitative assessment of tumor growth due to small sample size. Contrast agent ExiTron™ nano 12000 was injected intravenously 24 h before the first scan. Mice were anesthetized with 2% isoflurane and placed in 50 μm-resolution μCT scanner (Bruker SkyScan 1278). On the last day of study, C390-AuNC mice (1 for i. p. Group, 2 for oral group) were also injected with the contrast agent and scanned along with the disease control mice. A comparison of disease control vs C390-AuNC i. p. Treated mice livers (n = 1) is attached as a z-stack video for qualitative assessment of tumor growth.

##### 4.2.2.4 Protein expression

Protein expression of cytokines such as IFN-γ, IL-10, IL-6 and TNF-α (ELISA kit, K15048D Meso Scale Discovery, USA), vascular endothelial growth factor (VEGF) (ELISA kit, K15069M Meso Scale Discovery, USA), and TGF-β1 (ELISA kit, K151XWK, Meso Scale Discovery, USA) [48], was measured in tumor + non-tumor sections due to the difficulty of collecting complete necrotic tumors from the liver samples during the HCC study [53]. The data was plotted as protein concentration (pg/mL) and statistically analyzed.

### 4.3 Statistical analysis

Statistical analyses were conducted using GraphPad Prism 10 (CA, USA). The Grubbs test was utilized to identify and exclude outliers within each group. For multiple group comparisons, one-way ANOVA followed by Tukey's *post hoc* test was employed. For comparison of groups within and between different time points, two-way ANOVA followed by Tukey's *post hoc* test was employed. Differences between two groups were analyzed using the *t-*test. Results are presented as mean ± standard deviation (SD) except for data in Fig. 5(C&D) and SI(6&7) that are presented as mean ± standard error of the mean (SEM). A *p*-value of less than 0.05 was considered statistically significant and different ∗ refer to ∗p < 0.05, ∗∗p < 0.01, ∗∗∗p < 0.001, ∗∗∗∗p < 0.0001.

## Ethics approval and consent to participate

All animal studies described within the manuscript were approved by and performed in accordance with the local animal committee under reference 2024/UCL/MD/002.

## CRediT authorship contribution statement

**Tanya Saxena:** Conceptualization, Formal analysis, Investigation, Methodology, Visualization, Writing – original draft, Writing – review & editing. **Gabriela Guedes:** Conceptualization, Formal analysis, Investigation, Methodology, Visualization, Writing – original draft, Writing – review & editing. **Inês Domingues:** Investigation, Methodology. **Hafsa Yagoubi:** Investigation. **Léo Guilbaud:** Investigation. **William Van den Bossche:** Investigation. **Cristina Pangua:** Investigation. **Greetje Vande Velde:** Investigation, Methodology. **Bernard Ucakar:** Investigation. **Andrea García-Esnaola:** Investigation. **Isabelle Leclercq:** Formal analysis, Investigation, Methodology, Writing – review & editing. **Aitziber L. Cortajarena:** Conceptualization, Formal analysis, Funding acquisition, Investigation, Methodology, Project administration, Supervision, Visualization, Writing – review & editing. **Ana Beloqui:** Conceptualization, Formal analysis, Funding acquisition, Investigation, Methodology, Project administration, Supervision, Visualization, Writing – original draft, Writing – review & editing.

## Declaration of competing interest

The authors declare that they have no known competing financial interests or personal relationships that could have appeared to influence the work reported in this paper.
