# Supplementary material for: Engineered protein nanoclusters reduce liver fibrosis and hepatocellular carcinoma in mice models
Source: Bioact Mater. 2026 May 27;65:14–27. doi: 10.1016/j.bioactmat.2026.05.038 (PMC13235279; doi:10.1016/j.bioactmat.2026.05.038)
Supplement: Multimedia component 1 [file mmc1.docx]

**Supporting information**


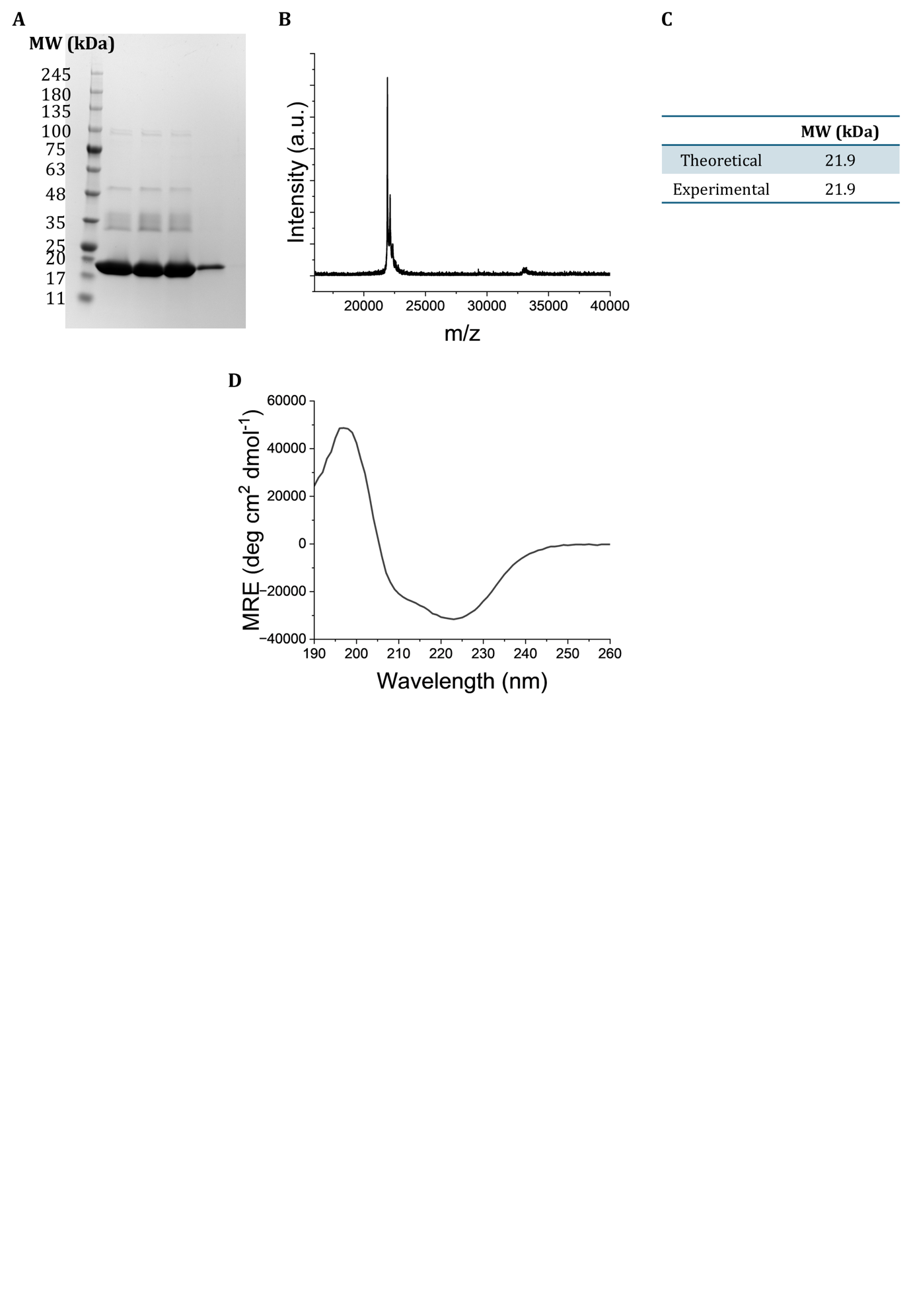


**Figure SI1.** Expression and purification of the protein scaffold. (A) SDS-PAGE of the purified protein scaffold. The different lanes contain samples obtained in different expression and purification batches. The first lane corresponds to the molecular weight marker, with the corresponding molecular weights (MW) displayed on the left. (B) MALDI-TOF of the purified protein. (C) Comparison between the theoretical (calculated based on the protein sequence on ProtParam) and the experimentally obtained molecular weights. (D) Circular dichroism spectrum of the purified protein scaffold.


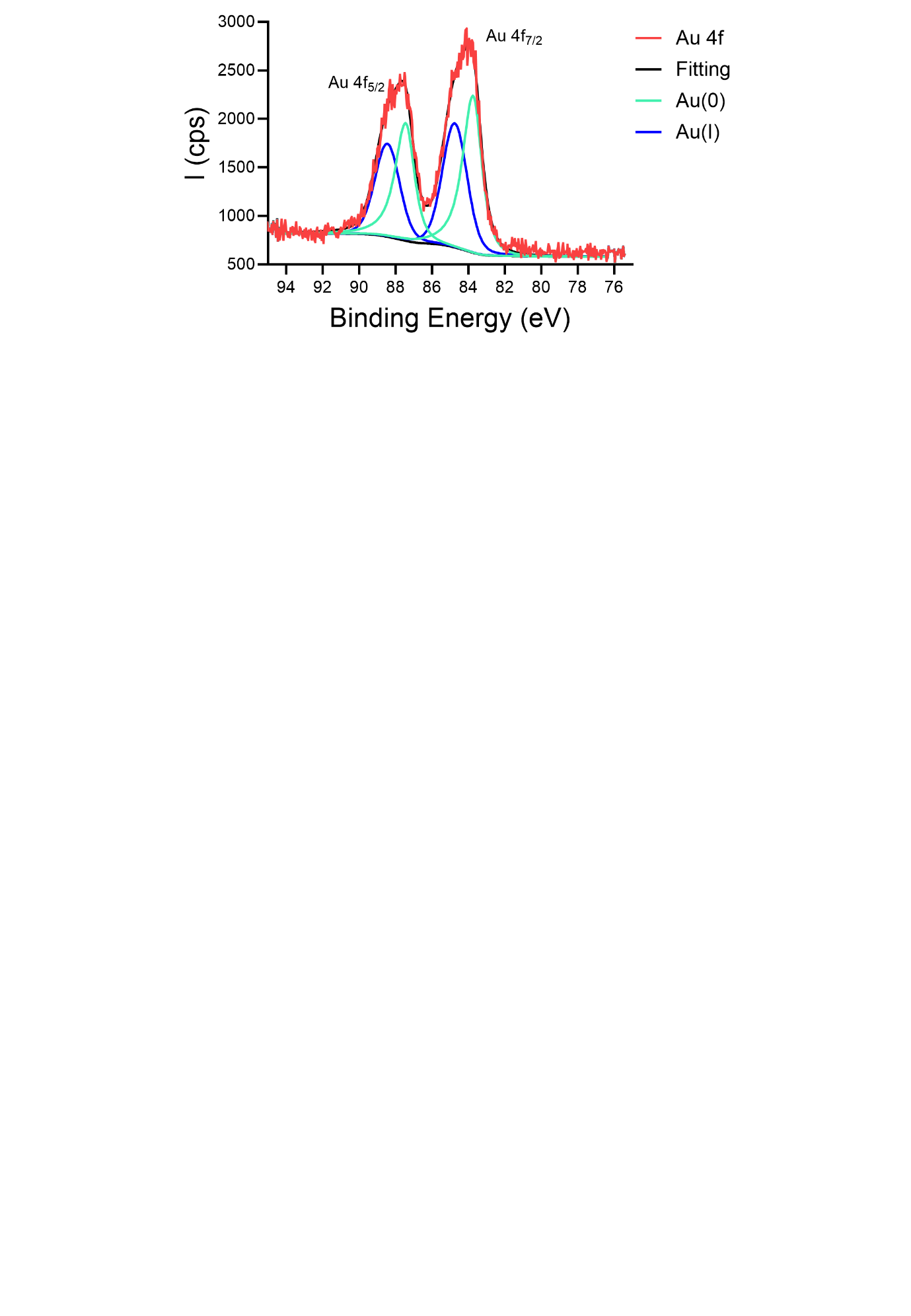


**Figure SI2.** X-ray photoelectron spectroscopy (XPS) spectrum of C390-AuNCs with respective fitting and deconvolution of the Au 4f peaks. The measured spectrum is shown in red, the overall fit in black, and the deconvolution of the Au(0) and Au(I) components in green and blue, respectively.


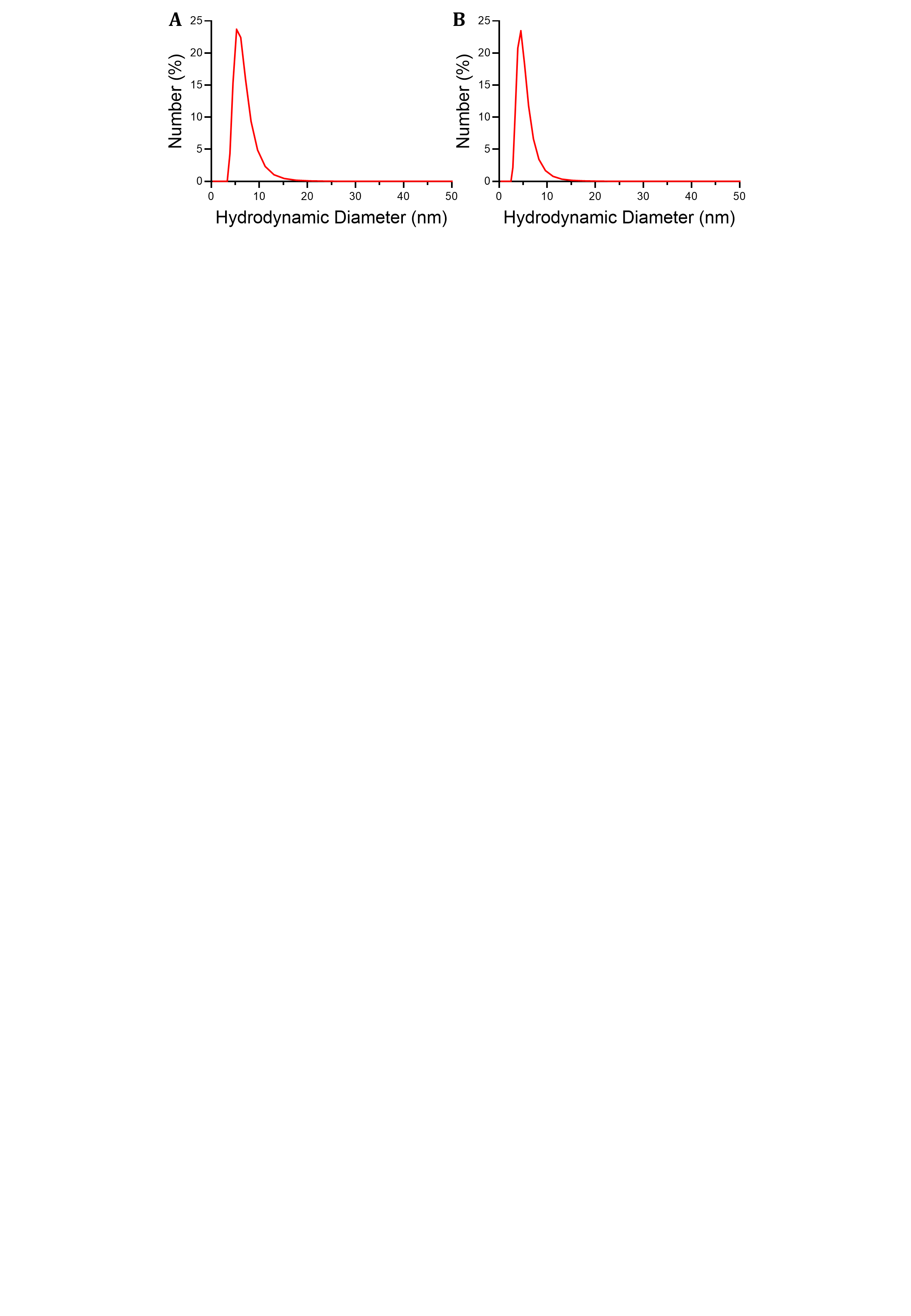


**Figure SI3.** Hydrodynamic diameter distributions of C390-AuNCs measured by Dynamic Light Scattering (DLS) in (A) PBS and (B) Mili-Q water.


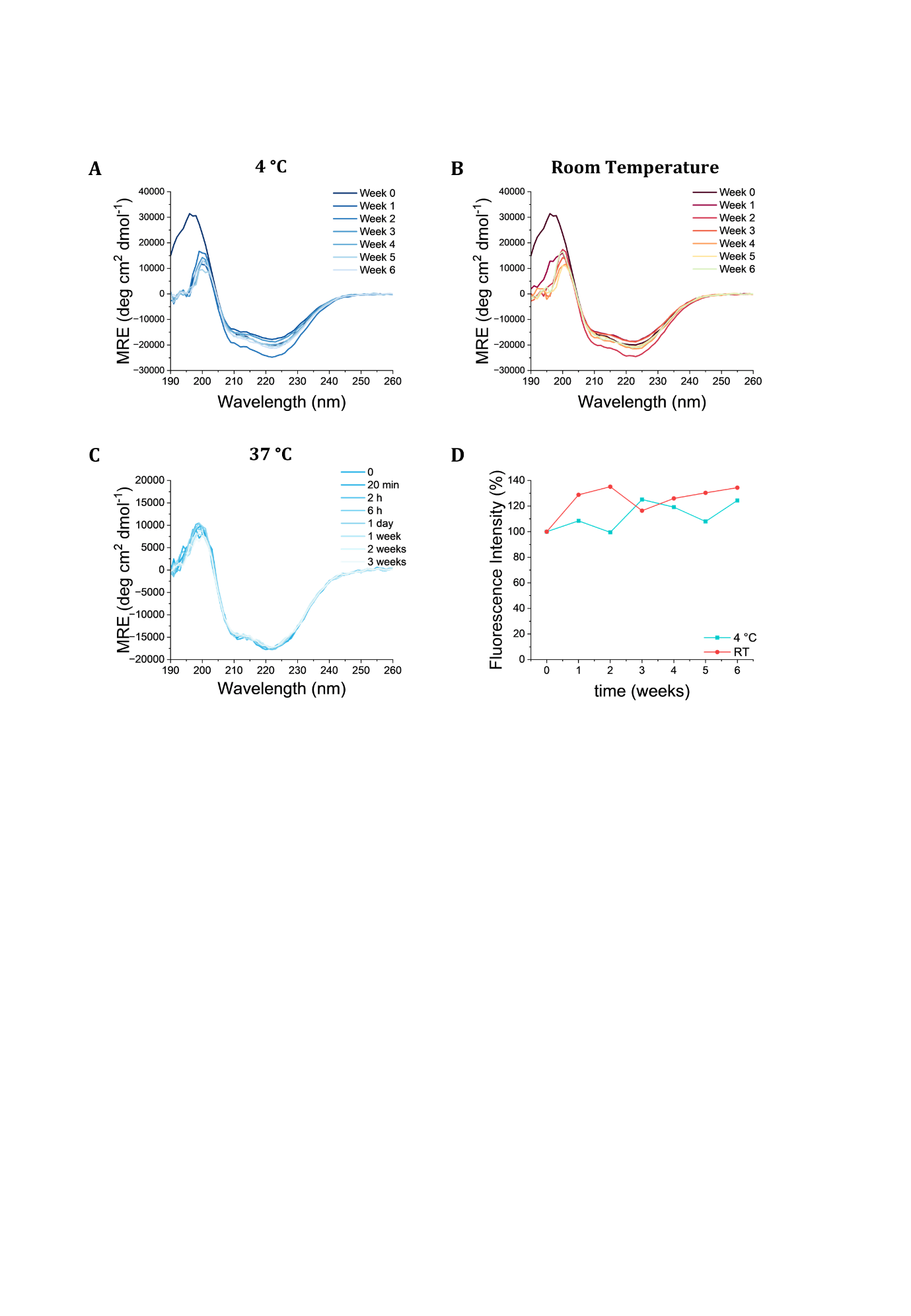


**Figure SI4.** Stability of C390-AuNC under storage or physiological conditions. (A) Circular dichroism spectra of the C390-AuNC stored at 4 ºC, (B) at room temperature, or (C) at 37 ºC. (D) Fluorescence intensity of the C390-AuNC stored at 4 ºC (blue points and line) or at room temperature (red points and line).


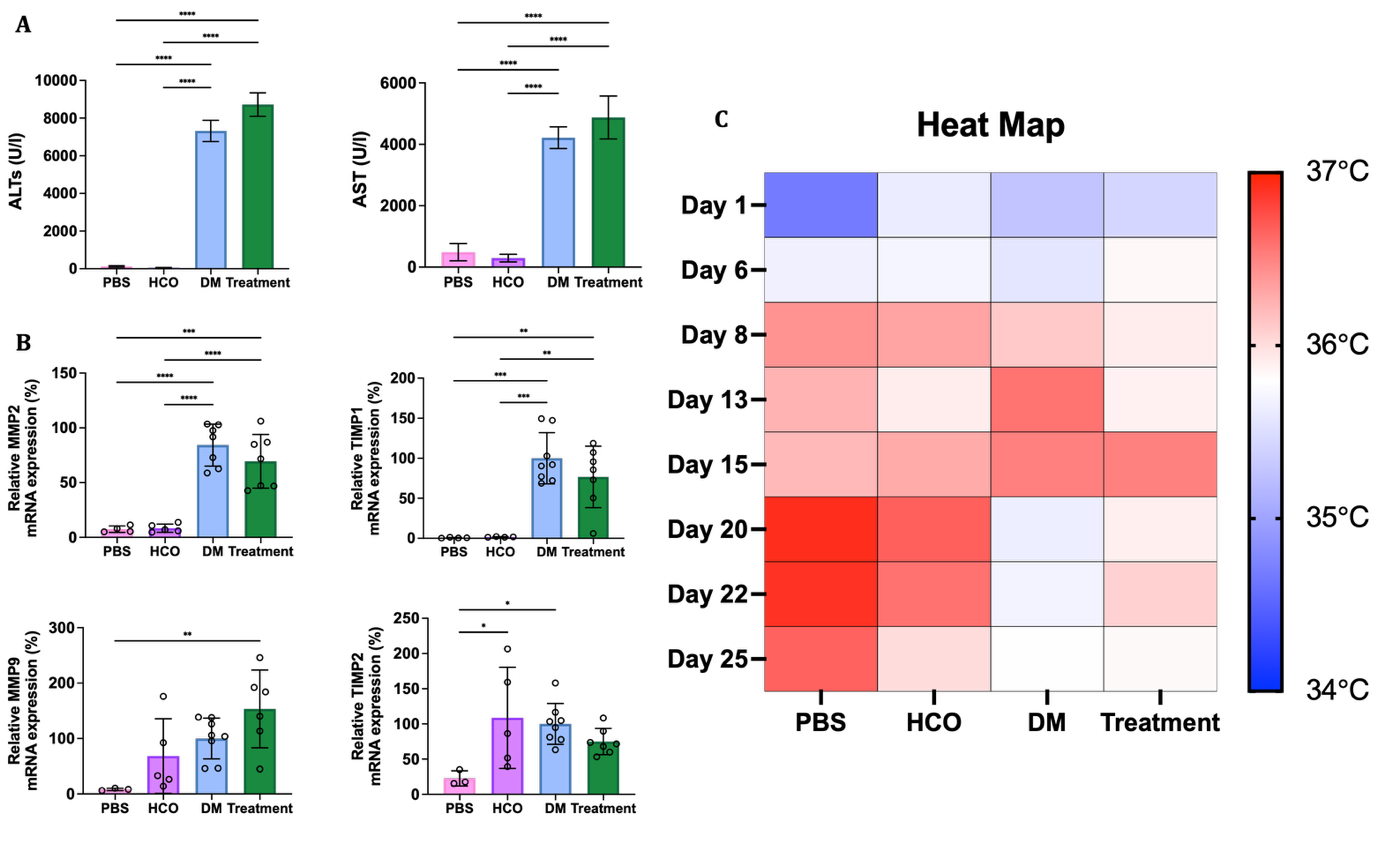


**Figure SI5.** Liver toxicity, mRNA expression and real-time thermal imaging of livers during liver fibrosis study. (A) ALT and AST levels in plasma were measured after sacrificing mice, (B) Relative mRNA expression of genes MMP2, MMP9, TIMP1 and TIMP2 were measured using qPCR to quantify degradation of collagen fibers, and (C) Heat map showing real-time thermal energy emissions from liver demonstrating inflammation and liver homeostasis. Data points shown as Mean$\pm$SD, n=5-8, Statistical analysis: One-way ANOVA followed by Tukey’s post hoc test (*p < 0.05, **p < 0.01, ***p< 0.001, and ****p<0.0001).


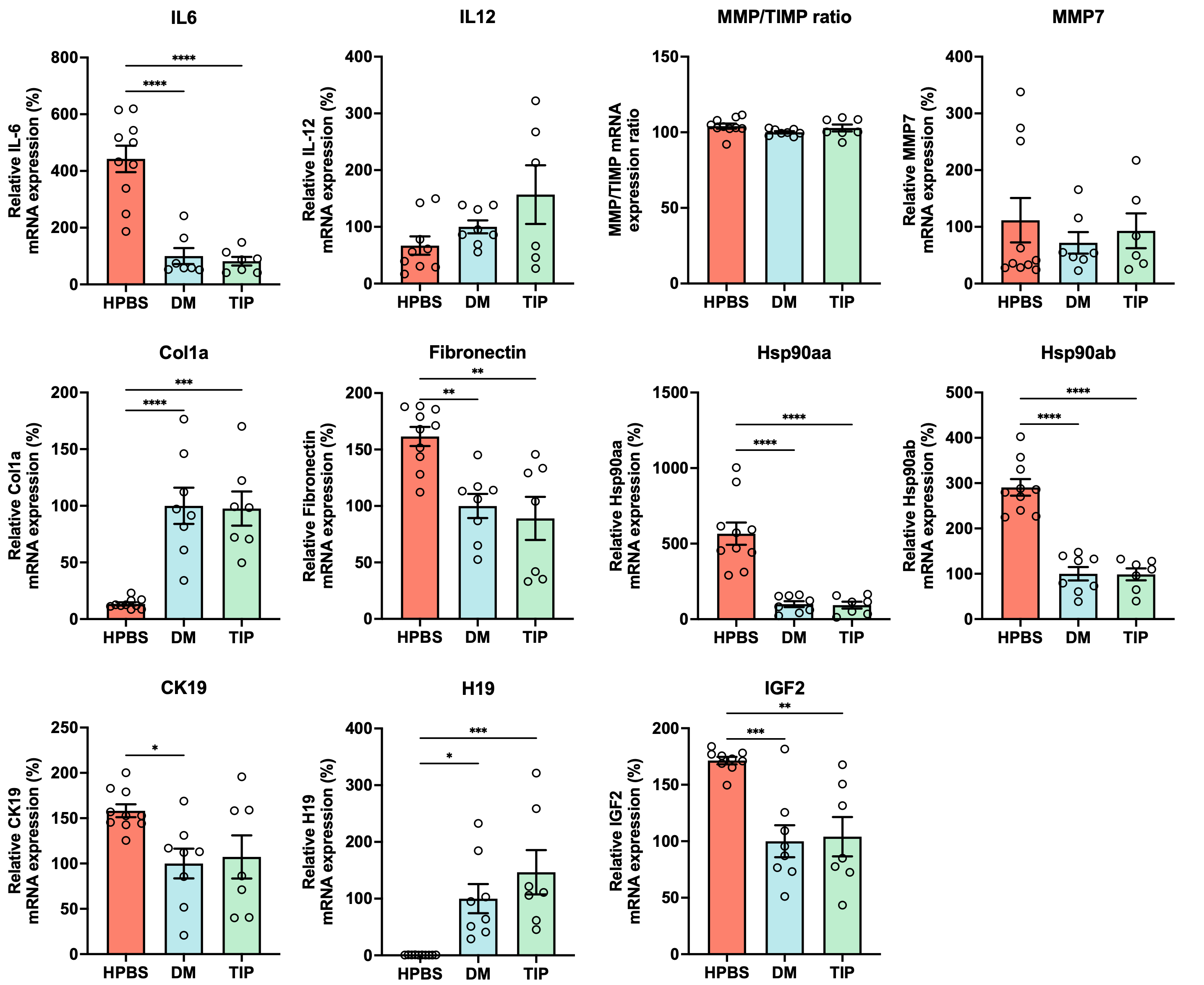


**Figure SI6.** Relative mRNA expression of fibrotic markers (Col1a, fibronectin & MMP/TIMP ratio), Hsp90 expressing genes (Hsp90aa & Hsp90ab), cancer markers (MMP7, CK19, H19 & IGF2) and inflammatory markers (IL6 & IL12) measured using qPCR (n=7 to 10, statistical analysis: One-way ANOVA Tukey’s test, Data points shown as Mean±SEM, *p < 0.05, **p < 0.01, ***p< 0.001, and ****p<0.0001).

**
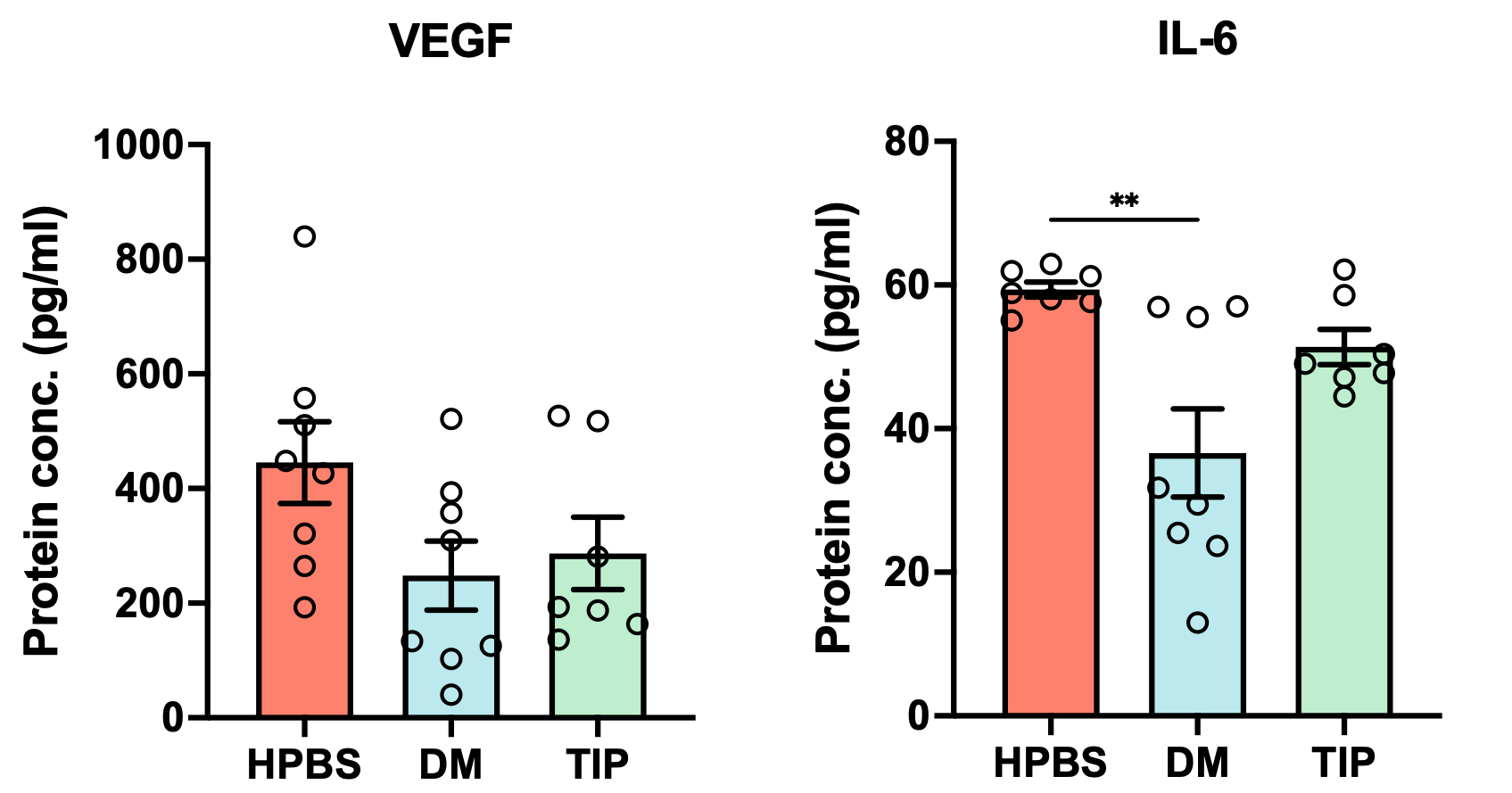
**

**Figure SI7.** Protein expression of VEGF and IL-6 measured by ELISA in liver samples for the hepatocellular carcinoma *in vivo* study (n=7 to 10, statistical analysis: One-way ANOVA Tukey’s test, Data points shown as Mean±SEM, **p < 0.01).
